# Supplementary material for: Content-rich biological network constructed by mining PubMed abstracts
Source: BMC Bioinformatics. 2004 Oct 8;5:147. doi: 10.1186/1471-2105-5-147 (PMC528731; doi:10.1186/1471-2105-5-147)
Supplement: Additional File 2 — The original results of the above study (non-essential files are deleted to keep the file size under the limit set by BMC bioinformatics). [file 1471-2105-5-147-S2.bz2 › chilibotAdditionalFile2/dip30/48ID7824954E190/html/VP16_GTF2B.html]

 


 **VP16** and **GTF2B** 
  
Found 65 abstracts in PubMed, retrieved 30.  
 

 What does Google say? 
 PDF only 
| .edu only 

---

**Interactive relationship** (e.g. stimulation, inhibition, etc)

**Neutral relationship**- Characterization of the interaction between the acidic activation domain of  **VP16**  and the RNA polymerase II initiation factor TFIIB  [ **GTF2B** ] .  Ref: 8710503 Nucleic Acids Res, 1996
- In this report we have used several strategies to critically assess this interaction between  **VP16**  and TFIIB  [ **GTF2B** ] .  Ref: 8710503 Nucleic Acids Res, 1996
- Human general transcription factor TFIIB  [ **GTF2B** ]  conformational variability and interaction with  **VP16**  activation domain.  Ref: 9609687 Biochemistry, 1998
- TFIIB  [ **GTF2B** ]  interacts with both pX and  **VP16**  simultaneously.  Ref: 8670843 EMBO J, 1996
- In assays of direct binding,  **VP16**  bound recombinant human TFIIB  [ **GTF2B** ]  but not Drosophila or yeast TFIIB  [ **GTF2B** ] .  Ref: 8710503 Nucleic Acids Res, 1996
- Moreover, a direct comparison of domains within human TFIIB  [ **GTF2B** ]  hTFIIB required for  **VP16**  and BPV 1 E2 indicates that these acidic activators interact with hTFIIB in a qualitatively similar manner.  Ref: 9311902 J Virol, 1997
- Taken together our results suggest more evidence is needed to support the notion that TFIIB  [ **GTF2B** ]  is a functionally important target for the activator  **VP16** .  Ref: 8710503 Nucleic Acids Res, 1996
- This result implies DNA bending and looping of promoter DNA as a result of the physical interaction between GAL4  **VP16**  and an interface of the TBP TFIIA TFIIB  [ **GTF2B** ]  complex.  Ref: 12538582 J Biol Chem, 2003
- In the transient repression experiment, a region containing 34 amino acids of the activation domain of  **VP16**  412 456 which interacts with TFIIB  [ **GTF2B** ]  was found to be necessary and sufficient for the tTA mediated repression of gene expression.  Ref: 9875451 Arch Pharm Res, 1998
- Here, we describe mutations on the DNA binding surface of TFIIB  [ **GTF2B** ]  that severely affect both TATA binding protein TBP TFIIB  [ **GTF2B** ]  TATA complex formation and interaction with the  **VP16**  activation domain in vitro.  Ref: 9372910 Mol Cell Biol, 1997
- We show that the  **VP16**  activation domain directly interacts with TATA binding protein TBP, TFIIB  [ **GTF2B** ] , and the SAGA histone acetylase complex in vivo.  Ref: 12297514 J Biol Chem, 2002
- The hybrid TAF did not mediate activation by  **VP16**  or vitamin D receptor, each of which interacts with TFIIB  [ **GTF2B** ] , but not with dTAF II 110 amino acid 1 308.  Ref: 11687654 Proc Natl Acad Sci U S A, 2001
- The 14 3 3 TFIIB  [ **GTF2B** ]  interaction appears to occur within the same domain of TFIIB  [ **GTF2B** ]  that binds the human herpes simplex virus transcriptional activator  **VP16** , because  **VP16**  and 14 3 3 were able to compete for interaction with TFIIB  [ **GTF2B** ]  in vitro.  Ref: 10449590 Plant Cell, 1999
- From 1982 to 1997 thirty six previously untreated patients with advanced HD, Stages IIB  [ **GTF2B** ]  to IV according to the Ann Arbor classification, were treated with the ChlVPP ABV  **VP16**  regimen.  Ref: 10221511 Leuk Lymphoma, 1999
- Site specific protein DNA photo cross linking was used to show that, when bound to its cognate site at various distances upstream of the TATA element, the chimeric transcriptional activator GAL4  **VP16**  can physically interact with a TATA box binding protein TBP transcription factor IIA TFIIA TFIIB  [ **GTF2B** ]  complex assembled on the TATA element.  Ref: 12538582 J Biol Chem, 2003
- TFIIB  [ **GTF2B** ]  is composed of two domains which are engaged in an intramolecular interaction that is disrupted upon interaction with the activation domain of the Herpesvirus  **VP16**  protein in vitro 2 3.  Ref: 10712906 Curr Biol, 2000
- Affinity columns of  **VP16**  bound TFIIB  [ **GTF2B** ]  activity from HeLa cell extracts and the binding was reduced by mutations in the activation domain of  **VP16** .  Ref: 8710503 Nucleic Acids Res, 1996
- Transcription rates can be enhanced by activator proteins, and the classical example is the herpes simplex virus factor  **VP16**  alpha TIF  [ **VP16** ] , which associates with cellular transcription factors, including TFIIB  [ **GTF2B** ] .  Ref: 11371188 Biochemistry, 2001
- Repression assays revealed that WT1 represses all three classes of activation domains Sp1 and CTF, which stimulate initiation type I, human immunodeficiency virus type I Tat fused to a DNA binding domain, which stimulates predominantly elongation type IIA, and  **VP16** , p53 and E2F1, which stimulate both initiation and elongation type IIB  [ **GTF2B** ] .  Ref: 10390530 Nucleic Acids Res, 1999

**Non-interactive relationship** (e.g. studied together, co-existance, homology, etc.)

- We show that pX interacts with general transcription factors TFIIB  [ **GTF2B** ]  and TFIIH, as well as with the potent activation domain of  **VP16** .  Ref: 8670843 EMBO J, 1996
- Unlike binding from an extract we found that the interaction between  **VP16**  and recombinant human TFIIB  [ **GTF2B** ]  was not affected by mutations in  **VP16**  that reduce transactivation.  Ref: 8710503 Nucleic Acids Res, 1996
- Using order of addition and template challenge assays performed in a human cell free transcription system reconstituted with recombinant general transcription factors TFIIB  [ **GTF2B** ] , TBP, TFIIE, TFIIF, a recombinant general cofactor PC4, and highly purified epitope tagged multiprotein complexes TFIID, TFIIH, pol II, we demonstrate that when TBP is used as the TATA binding factor transcriptional activators such as Gal4  **VP16**  and human papillomavirus E2 mainly function by facilitating pol II entry to the promoter region.  Ref: 11457828 J Biol Chem, 2001
- Several different molecular targets have been suggested for direct contact by herpes simplex virus virion protein  **VP16** , including the general initiation factor TFIIB  [ **GTF2B** ] .  Ref: 8710503 Nucleic Acids Res, 1996
- Interestingly, these TFIIB  [ **GTF2B** ]  derivatives support viability, and they efficiently respond to Gal4  **VP16**  and natural acidic activators in different promoter contexts.  Ref: 9372910 Mol Cell Biol, 1997
- Point mutations within human TFIIB  [ **GTF2B** ]  that reduce transactivation by  **VP16**  have been shown to reduce  **VP16**  binding, but we show here that these same mutations critically affect both the important TBP TFIIB  [ **GTF2B** ]  interaction and the ability of TFIIB  [ **GTF2B** ]  to support activator independent basal transcription in vitro.  Ref: 8710503 Nucleic Acids Res, 1996
- The molecular shapes of transcription factors TFIIB  [ **GTF2B** ]  and  **VP16**  have been studied by small angle X ray scattering SAXS.  Ref: 11371188 Biochemistry, 2001
- Molecular shapes of transcription factors TFIIB  [ **GTF2B** ]  and  **VP16**  in solution implications for recognition.  Ref: 11371188 Biochemistry, 2001
